# Effectiveness and safety of cemiplimab in locally advanced and metastatic cutaneous squamous cell carcinoma

**DOI:** 10.3389/fphar.2026.1601650

**Published:** 2026-03-06

**Authors:** Gabriele Roccuzzo, Eleonora Bongiovanni, Giovanni Actis-Giorgetto, Chiara Astrua, Matteo Giovanni Brizio, Giovanni Cavaliere, Paolo Fava, Simone Ribero, Pietro Quaglino

**Affiliations:** Section of Dermatology, Department of Medical Sciences, University of Turin, Turin, Italy

**Keywords:** cemiplimab, checkpoint inhibitor therapy, cutaneous squamous cell carcinoma, immunosuppression, PD-1 inhibitors, real-life

## Abstract

Cutaneous squamous cell carcinoma (cSCC) is a common skin cancer with increasing incidence. The anti-PD-1 therapy cemiplimab has shown its antitumor activity in locally advanced (lacSCC) and metastatic cSCC (mcSCC). This retrospective study assessed the real-life effectiveness and safety of cemiplimab in 83 patients with lacSCC (n = 53) and mcSCC (n = 30). The objective response rate (ORR) was 49.4%, with a complete response (CR) in 15.7% and a partial response (PR) in 33.7%. The median progression-free survival (PFS) was 14 months (95% CI 9–55) and the median overall survival (OS) 19 months (95% CI 10–39). Half of patients (50.6%) experienced adverse events (AE) of any grade, with 8.4% discontinuing therapy due to the severe AEs. The subset of patients who experienced progression during therapy displayed younger age (p = 0.002), a higher disease stage at baseline (p = 0.003), and a nodal disease (p = 0.041). No differences in survival outcome emerged between patients with nodal vs. distant metastases, previous radiotherapy recipient vs. radiotherapy-naïve, and immunosuppressed vs. immunocompetent patients. Head&neck tumor site was associated with a longer OS after first progression (OS2, HR 0.29, 95% CI 0.09–0.89). This study supports the safe and effective use of cemiplimab in real life clinical practice yet highlights the need for further identification of new predictors of clinical response.

## 1 Introduction

Cutaneous squamous cell carcinoma (cSCC) is one of the most common keratinocyte-derived cancers deriving from the malignant proliferation of epidermal keratinocytes (Nagarajan et al., 2019). Its worldwide incidence is constantly increasing and nowadays accounts for 20% of all cutaneous malignancies (Zakhem et al., 2023). In Europe, an age-standardized incidence of 9–96 cases per 100.000 for males and 5–68 cases per 100.000 for females has been described (Que et al., 2018). The etiopathogenesis of cSCC involves several factors, including exposure to ultraviolet (UV) radiation, advanced age, male sex, immunosuppression, human papillomavirus infection, smoking, and genetic factors (Rundel, 1983; Chahoud et al., 2016; Conforti et al., 2019; Pirie et al., 2018; Dusingize et al., 2017). The role of UV radiation in the pathogenesis of cSCC is supported by its onset on sun-exposed areas (mainly the headandneck area) in the context of multiple actinic keratoses in elderly patients with a clinically objectifiable UV-induced cutaneous damage (Alam and Ratner, 2001). Based on the disease extension and features, cSCC is classified into common primary and advanced cSCC. The latter group encompasses locally advanced cSCC (lacSCC), defined as non-metastatic cSCC not amenable to either surgery or radiotherapy with reasonable hope for cure, and metastatic cSCC (mcSCC), further classified as locoregional metastatic and distant metastatic cSCC (Stratigos et al., 2023). Although common primary cSCCs can be easily managed with surgery and have an excellent prognosis (5-year cure rates and 10-year overall survival exceeding 90%), patients with advanced cSCC face poorer outcomes (Brougham et al., 2012). Historical data on the efficacy of off-label chemotherapy regimens showed median progression-free survival and disease-free survival of 6 and 14.6 months, respectively, with short-lived responses and significant toxicity (Chapalain et al., 2020; Trodello et al., 2017). More recently, thanks to its high tumor mutational burden and subsequent expression of neoantigens on tumor cells, immunotherapy with immune-checkpoint inhibitors (ICI) has represented a breakthrough in the management of advanced cSCC (In et al., 2021; Salzmann et al., 2020). Specifically, cemiplimab was the first monoclonal antibody targeting the programmed cell death protein 1 (PD-1) receptor approved in 2018 by the Food and Drug Administration and in 2019 by the European Medicines Agency for patients with lacSCC and mcSCC not eligible for curative surgery or radiation. By preventing the binding of PD-1 receptors on T-cells to PD-L1-expessing neoplastic cells, cemiplimab can promote an anti-tumor activity boosting the immune response. In terms of efficacy and safety, the final data from the different treatment arms of the phase II clinical trial EMPOWER were published in early 2025. At 42.5 months, the objective response rate (ORR) for groups 1–2 (3 mg/kg every 2 weeks) and 3 (350 mg every 3 weeks) was 47.2%, with an estimated 12-month duration of response of 88.3% and a median PFS of 26.0 months. Serious treatment-emergent adverse event rates were recorded in 31.1% (Hughes et al., 2025). Moreover, real-world data have been suggesting different effectiveness trends based on some clinical features, with stronger responses in patients with headandneck cSCC tumors and worse outcomes in cases of genital involvement, low performance status, prior nodal radiation therapy or chemotherapy (In et al., 2021; Baggi et al., 2021; Haigh et al., 2025). In this context, real-world data on the activity and safety of ICIs, capturing a broader patient population often excluded from clinical trials, are essential for clinicians to make informed decisions and optimize therapy selection. Moreover, the assessment of strong clinical-pathological predictors of response have yet to be defined. This study stands as a real-world analysis, with the aim to provide clinicians with new evidence on the safety and the effectiveness of cemiplimab in clinical practice.

## 2 Methods

A retrospective series of advanced cSCC patients treated with cemiplimab at the Dermatology Clinic of the Turin University Hospital, Italy, between August 2019 and February 2025 was collected. All patient information was sourced from the hospital’s database and archived within an internal computerized database. Patient inclusion criteria were: age >18, a histologically confirmed diagnosis of lacSCC or mcSCC, a minimum of 2 treatment infusions at standard dose (350 mg every 3 weeks), and the presence of complete medical records. Cemiplimab was administered until disease progression, death, unacceptable toxicity, or patient choice. The primary endpoints were to investigate cemiplimab clinical activity in terms of response rate and safety profile. Progression-free survival (PFS) was defined as the time from the start of therapy to the date of the first progression or death from any cause, overall survival (OS) as the time from the start of therapy until death, and overall survival 2 (OS2) as the time from first progression to death after censoring. The clinical response to cemiplimab was evaluated according to the RECIST (Response Evaluation Criteria in Solid Tumors) criteria version 1.1 (Eisenhauer et al., 2009). Best response rates were defined as complete response (CR), partial response (PR), stable disease (SD) and progressive disease (PD). Objective response rate (ORR) was recorded from the start of the study treatment until the end of treatment as the proportion of CR and PR observed. Disease control rate (DCR) also included patients with SD. The secondary objective was the identification of any clinical or pathological characteristics associated with better clinical outcomes. The following clinical-pathological features were analyzed: gender, age, presence of immunosuppressive disorders or skin comorbidities, date of diagnosis of advanced cSCC, tumor site, histological grading, presence of lymphovascular and perineural invasion, staging according to the “Tumor, Lymph Nodes, Metastasis” (TNM) classification system (8th edition), previous or subsequent treatments (surgery, chemotherapy, radiotherapy), type of progression (local, loco-regional, distant metastasis), date of initiation and discontinuation of treatment with cemiplimab, reason for discontinuation, best response achievement, and treatment-related toxicity assessed according to the Common Terminology Criteria for Adverse Events (CTCAE) version 5.0 (Gershenwald et al., 2017; Freites-Martinez et al., 2021). Grade 3, 4, or 5 AEs were considered severe (Freites-Martinez et al., 2021). Descriptive statistics were applied to analyze patient and tumor characteristics. The proportional hazards assumption based on Schoenfeld residuals was tested after fitting the Cox models. Survival curves were generated using the Kaplan-Meier method and analyzed with the Log-rank test. For patients alive without disease progression nor death, data were censored on the date of last patient contact. A p-value of ≤0.05 was considered statistically significant. All statistical analyses were performed using Stata/SE.v.17 Software (StataCorp, College Station, TX).

## 3 Results

### 3.1 Patients’ and disease characteristics

A total of 83 patients were included in the analysis. Their baseline characteristics are summarized in Table 1. Overall, most patients were male (n = 55, 66.3%), with a median age at diagnosis of advanced cSCC of 78 years (range 19–93). LacSCC accounted for 53 (63.9%) patients, whilst mcSCC for 30 cases (36.1%). An immunodeficiency of any kind was reported in 19 patients (22.9%), as follows: 5 non-Hodgkin lymphoma, 3 chronic lymphocytic leukemia, 3 bullous epidermolysis, 2 kidney transplant, 1 xeroderma pigmentosum, 1 Epidermodysplasia Verruciformis, 1 marginal B-cell lymphoma, 1 chronic autoimmune hepatitis, 1 VEXAS syndrome, and 1 lichen planus requiring systemic therapy. According to baseline TNM staging, 52 patients (64.2%) had a T grade >2, and 57 (68.7%) had an N grade ≥1. As for histological features, poor differentiation (G3), perineural invasion, and lymphovascular invasion were detected in 17 (20.5%), 45 (72.6%) and 54 (83.1%) patients, respectively. Previous treatment of the primary tumor included surgical excision in 61 patients (73.5%). Additionally, 42 patients (50.6%) received radiotherapy (RT), which was performed at the primary tumor area in 34 cases (80.9%), at regional lymph node metastases in 5 patients (11.9%), and at both areas in 3 cases (7.1%). In total, 36 patients (43.4%) experienced disease progression. Progression sites were not mutually exclusive and included 26 (31.3%) local progressions, 16 (19.2%) nodal spreads, and 7 (8.4%) distant metastases. Death occurred in 41 patients (49.4%).

**TABLE 1:** Study population.

Baseline Cohort (n = 83)			
Patient characteristics		No (%)	
Median age - years (range)		78 (19–93)	
Male sex		55 (66.3%)	
Female sex		28 (33.7%)	
Immune deficiency		19 (22.9%)	
Ongoing immunosuppressive treatments		​	
Everolimus 1–2 mg/d		2	
Prednisone 0.25 mg/kg/d		2	
VEXAS syndrome (Prednisone 12.5–25 mg/d)		1	
Lichen ruber planus (0.25 mg/kg/day + Dupilumab)		1	
Conditions associated with secondary immune impairment		​	
Xeroderma pigmentosum		1	
Epidermolysis bullosa		3	
Epidermodysplasia verruciformis		1	
History of immunosuppressive treatments		​	
Chronic lymphocytic leukemia (Ibrutinib)		3	
Non-Hodgkin lymphoma (R-CHOP)		5	
Autoimmune hepatitis (azathioprine)		1	
M0		53 (63.9%)	
M1		30 (36.1%)	
Histological grading		​	
Not evaluable		23 (27.7%)	
G1		9 (10.8%)	
G2		34 (40.9%)	
G3		17 (20.5%)	
Stage		​	
II		14 (16.8%)	
III		37 (44.6%)	
IVa		2 (2.5%)	
IVb		30 (36.1%)	
Perineural invasion		45 (72.6%)	
Lymphovascular invasion		54 (83.1%)	
Radiotherapy		42 (50.6%)	
Site of radiotherapy		​	
Primary lesion		34 (80.9%)	
Lymph nodes		5 (11.9%)	
Primary lesion + lymph nodes		3 (7.1%)	
Prior surgery		61 (73.5%)	
Chemotherapy after cemiplimab		7 (8.4%)	
Overall progression		36 (43.4%)	
Skin progression		26 (31.3%)	
Nodal progression		16 (19.2%)	
Distant metastases		7 (8.4%)	
Progression-free survival, months, median (95% CI)		14 (9–55)	
Overall survival, months, median (95% CI)		19 (10–39)	
​	Progressed (n = 36)	Not progressed (n = 47)	p-value*
Age, median (range)	74 (19–86)	80 (50–93)	**0.002**
Male sex, n (%)	25 (69.4)	30 (63.8)	0.592
Stage, n (%)	II: 1 (2.7) III: 22 (61.1) IVa: 1 (2.8) IVb: 12 (33.3)	II: 13 (27.7) III: 15 (31.9) IVa: 1 (2.1) IV: 18 (38.3)	**0.003**
Head and Neck, n (%)	20 (55.5)	29 (61.7)	0.573
N+, n (%)	29 (80.5)	28 (59.6)	**0.041**
M+, n (%)	12 (30.3)	18 (38.3)	0.463
Grading, n (%)	G1: 4 (11.1) G2: 13 (36.1) G3: 6 (16.7)	G1: 5 (10.4) G2: 21 (43.8) G3: 11 (22.9)	0.752 0.799 0.665
Perineural invasion, n (%)	19 (52.8)	26 (55.3)	0.243
Lymph vascular invasion, n (%)	28 (77.8)	26 (55.3)	0.137
Immune suppression, n (%)	6 (16.7)	13 (27.6)	0.237
Radiotherapy, n (%)	18 (50.0)	24 (51.0)	0.923
Toxicity of any type, n (%)	15 (41.7)	27 (57.4)	0.154
Months to objective response, mean (sd)	3.7 (0.5)	6.1 (1.0)	**0.032**
Type of best objective response, n (%)	CR: 1 (2.7) PR: 8 (22.2) SD: 8 (22.2) PD: 19 (52.8)	CR: 12 (25.6) PR: 20 (42.6) SD: 15 (31.9) PD: 0 (0.0)	**<0.001**

*Statistically significant values are depicted in bold font.

### 3.2 Effectiveness of treatment

**FIGURE 1:**
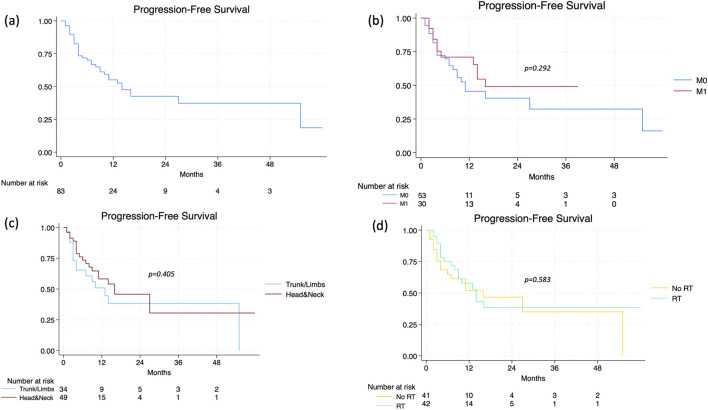
**(a)** Progression-free survival (PFS) in the overall population; **(b)** according to the presence of distant metastases; **(c)** according to primary tumor site; **(d)** according to previous radiotherapy.

**FIGURE 2:**
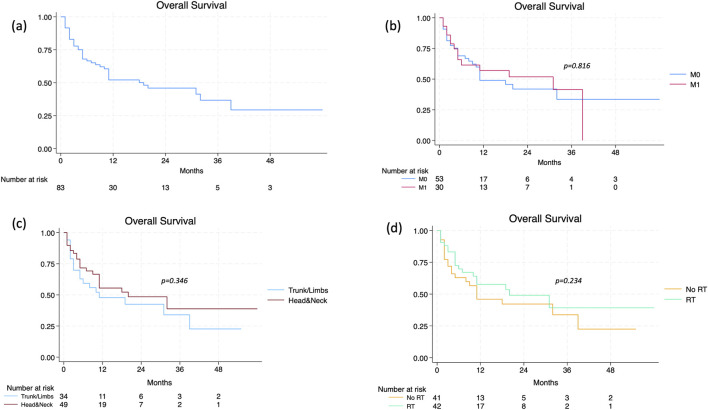
**(a)** Overall survival (OS) in the overall population; **(b)** according to the presence of distant metastases; **(c)** according to primary tumor site; **(d)** according to previous radiotherapy.

**FIGURE 3:**
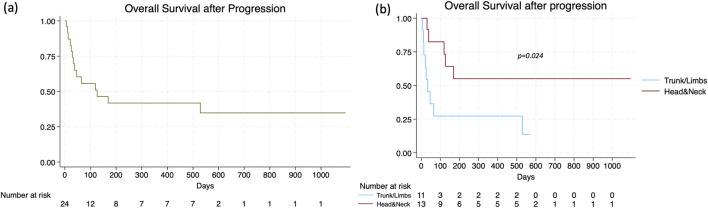
**(a)** Sub-analysis of patients with progressive disease without death as the primary event; **(b)** OS2 after progression according to primary tumor site (head and neck vs trunk/limbs).

At the time of data analysis, treatment with cemiplimab was ongoing in 10 patients (12.0%), whilst 73 (88.0%) patients had discontinued it. The median duration of treatment was 5 months (range 1–59), with a median number of doses of 7 (range 1–78). The best ORR was 49.4% (95% CI 38.2–60.6), with CR in 13 patients (15.7%) and PR in 28 patients (33.7%); SD was observed in 23 patients (27.7%), whilst PD in 19 patients (22.9%). The recorded DCR was 77.1% (95% CI 65.3–84.6). A median of 3 months (range 1–29) elapsed from the start of cemiplimab to objective response achievement. The subset of patients who experienced progression during therapy displayed younger age (p = 0.002), a higher disease stage at baseline (p = 0.003), and a nodal disease (p = 0.041). The median PFS was recorded at 14 months (95% CI 9–55), with no significant differences between lacSCC vs. mcSCC patients (p = 0.292), headandneck vs. trunk/limb location (p = 0.405), and RT-pretreated vs. RT-naïve patients (p = 0.583) (Figure 1). The median time to progression was significantly longer in patients achieving a CR (not reached), compared to those reaching PR (16 months, 95% CI 11-NA) and SD (11 months, 95% CI 4-NA). Regarding OS, the median value stood at 19 months in the entire population (95% CI 10–39). The presence of a metastatic disease at baseline (p = 0.816), as well as the headandneck location (p = 0.346) and a prior radiation therapy (p = 0.234) were not significantly related to worse outcome (Figure 2). Patients who achieved CR had a median OS of 32 months (95% CI 32-NA), compared to those who reached PR (20 months, 95% CI 10-NA), SD (6 months, 95% CI 2-NA), or PD (4 months, 95% CI 3–11) (p < 0.001). A significant difference in terms of OS was highlighted based on the presence of a CR to cemiplimab or a PD. In Cox regression analysis, patients who achieved a CR had an 85% reduction in the risk of death, and a better OS, compared to those who did not achieve such a response (HR 0.15, [95% CI 0.04–0.64], p = 0.010). On the other hand, a PD implied a nearly threefold increase in the risk of death (HR 2.81, [95% CI 1.43–5.55], p = 0.003). The sub-analysis on OS2 after censoring (n = 24) pointed out the predictive relevance of age and headandneck location in this subset of patients. Indeed, an older age determined a 5% increase in the risk of death (HR 1.05, [95% CI 1.01–1.11], p = 0.049), while headandneck location a 71% reduction in that risk (HR 0.29, [95% CI 0.09–0.89], p = 0.031), with a median OS2 after progression of 126 days (95% CI 36-NA) (Figure 3).

### 3.3 Safety of treatment

Overall, 42 patients (50.6%, 95% CI 39.4–61.8) experienced at least one AE of any grade, with asthenia (35.7%), anemia (14.3%) and diarrhea (11.9%) being the most frequent, usually self-limiting, and manageable with supportive therapy only. In 13 cases (15.7%) the AE was defined as grades 3 or 4, with 4 patients (9.5%) experiencing severe anemia, 2 (4.8%) facing serious asthenia that impaired daily living activities, one case (2.4%) of hypocortisolism, and one patient (2.4%) that required hospitalization due to acute myocarditis. In 8.4% of patients the development of a severe AE justified treatment discontinuation, specifically three cases of G3 anemia, two cases of G3 asthenia, one case of severe diarrhea, a case of myocarditis, and one high-grade hypocortisolism. No fatal AEs were recorded (Supplementary Figures S1-S3).

## 4 Discussion

Phase II and III clinical trials take place in controlled settings and specialized centers, where treatments follow strict monitoring protocols to reduce variability and control influencing factors. While this ensures to achieve high-quality results, it can make the findings less applicable to everyday clinical practice. Hence, real-world studies are essential to provide evidence on how treatments work in routine care, especially in those subsets of patients excluded clinical trials (e.g., comorbid and immune suppressed populations). Our data confirm the previously described efficacy and safety of cemiplimab in advanced cSCC, while introducing some relevant findings to the field (In et al., 2021; Salzmann et al., 2020; Baggi et al., 2021; Haist et al., 2022; Mallardo et al., 2024). Firstly, the ORR observed in this study (49.4%) is in line with both clinical trial and real-world data. Baggi et al. reported an ORR of 58%, though without PFS or OS data (Baggi et al., 2021). Haist et al. reported an ORR of 48.6% in a smaller cohort of 39 patients, while Mallardo et al. observed a lower ORR of 37% (Haist et al., 2022; Mallardo et al., 2024). Our findings fall between these values, with CR in 15.6% and PR in 33.7% of patients.

Secondly, regarding PFS, we observed a median of 14 months, which shows some differences from previous reports (29 months in the German registry vs. 8.8 months in the Italian one) (Haist et al., 2022; Mallardo et al., 2024). Conversely, our results are comparable to the 14.7 months reported in Cohort 6 of the EMPOWER study, which employed the flat dosing regimen (Hughes et al., 2025). For OS, our median of 19 months is lower than the 27.9 months reported by Mallardo et al., whilst the median was not reached in the EMPOWER trial (Hughes et al., 2025; Mallardo et al., 2024). However, differences in median follow up times and in patients’ age at the time of enrolment are likely to influence such findings (e.g., median age of 71 years in the EMPOWER cohort 1 vs. 78 years in our study) (Hughes et al., 2025).

Thirdly, the investigation of potential predictors of improved outcomes yielded new insights compared to previous reports. For instance, our data did not show a significantly better or worse outcome in patients previously treated with radiation therapy nor confirmed a superior response in patients with headandneck cSCC (Lo Greco et al., 2024). Nevertheless, OS2 was longer in such patients, which may be explained by the availability of EGFR inhibitors as an approved therapeutic option in case of advanced headandneck cSCCs (Baggi et al., 2021; Haist et al., 2022; Mallardo et al., 2024). Another key finding is that immunocompromised patients did not show significantly worse outcomes, unlike previous reports (Haist et al., 2022). This may be partially due to differences in immunosuppression definition, with our study investigating only few transplanted patients and including most patients with hematological malignancies, limiting the comparability of studies. Additionally, our findings suggest that classic clinical and histopathological predictors of disease aggressive behavior, such as tumor grading and lymphovascular invasion, may play a limited role in advanced settings. In fact, once the disease reaches an advanced setting, intrinsic aggressiveness may outweigh the conventional prognostic markers used in primary treatment of cSCC (Roccuzzo et al., 2024). Finally, we did not observe significant differences in survival outcomes between patients with nodal and distant metastatic disease, reinforcing the evidence that the natural history of advanced cSCC differs from other skin cancers amenable to ICI therapy, such as melanoma (Farrow et al., 2021; Ribero et al., 2024).

Regarding AE of any grade, which occurred in 50.6% of patients, our results resemble the 42.7% reported by Baggi et al. and the 34.3% by Haist et al. (Baggi et al., 2021; Haist et al., 2022). A higher incidence of AE is otherwise described in the EMPOWER-CSCC-1 study, standing at 98.8% in the group 6 and at 99.5% in groups 1–3, probability due to differences in AE reporting systems (Hughes et al., 2025). Nevertheless, asthenia, diarrhea, anemia, and pruritus confirmed to be the most frequent AEs, whilst Grade 3–4 AEs were significantly less frequent in real-world settings compared to clinical trials. In our study, the incidence was 15.7%, similar to the 9.2% reported by Baggi et al. and 17.9% by Haist et al., whereas the EMPOWER-CSCC-1 trial reported 49.2% in groups 1–3 (Hughes et al., 2025; Baggi et al., 2021; Haist et al., 2022). The rate of treatment discontinuation due to AEs was 8.4% in our cohort, closely matching the 9.2% observed in a previous multicenter Italian study, and comparable to EMPOWER-CSCC-1 figures (13.9% in group 6% and 10.4% in groups 1–3) (Hughes et al., 2025; Baggi et al., 2021). Notably, no fatal AEs were observed in our study. In EMPOWER-CSCC-1, fatal AEs occurred in less than 10% of patients, and only two cases were reported by Baggi et al. (Hughes et al., 2025; Baggi et al., 2021). Despite the overall safety of cemiplimab, questions remain regarding the timing of potential therapy interruption. From a recent analysis, no difference has emerged in terms of OS, disease-specific survival and PFS between patients that discontinued treatment before censoring and those with standard treatment scheduled, suggesting that ICI treatment after 1 year might expose patients to further treatment-related events without advantages in effectiveness (Mallardo et al., 2024). As such, patients who have a response and are treated for 24 months may have durable responses, whilst there remains very limited evidence about safe therapy discontinuation before 12 months. In conclusion, our findings provide new evidence on the effectiveness and safety of cemiplimab in lacSCC and mcSCC, with some significant differences in the evaluated outcomes from the other previous real-life evidence. The challenge to identify clear and reproducible clinical, histological, and molecular predictors of response and their relationship with the duration of benefit is far from over.

## 5 Study limitations

Study limitations include its retrospective design, limited sample size with lack of control arm, absence of disease-specific mortality data and biomarker analysis, heterogeneity of the immunosuppressed subgroup, and the inclusion of only Caucasian patients. These limitations can potentially limit the generalizability of our findings.

## Data Availability

The data analyzed in this study is subject to the following licenses/restrictions: The collected data are not publicly available to protect patients’ privacy and comply with ethical requirements. Aggregated data supporting the study findings are available from the corresponding author upon a reasonable request. Requests to access these datasets should be directed to GR, gabriele.roccuzzo@unito.it.
