# Supplementary material for: Effectiveness and safety of cemiplimab in locally advanced and metastatic cutaneous squamous cell carcinoma
Source: Front Pharmacol. 2026 Mar 6;17:1601650. doi: 10.3389/fphar.2026.1601650 (PMC13002820; doi:10.3389/fphar.2026.1601650)
Supplement: Supplementary file 1 [file DataSheet1.pdf]

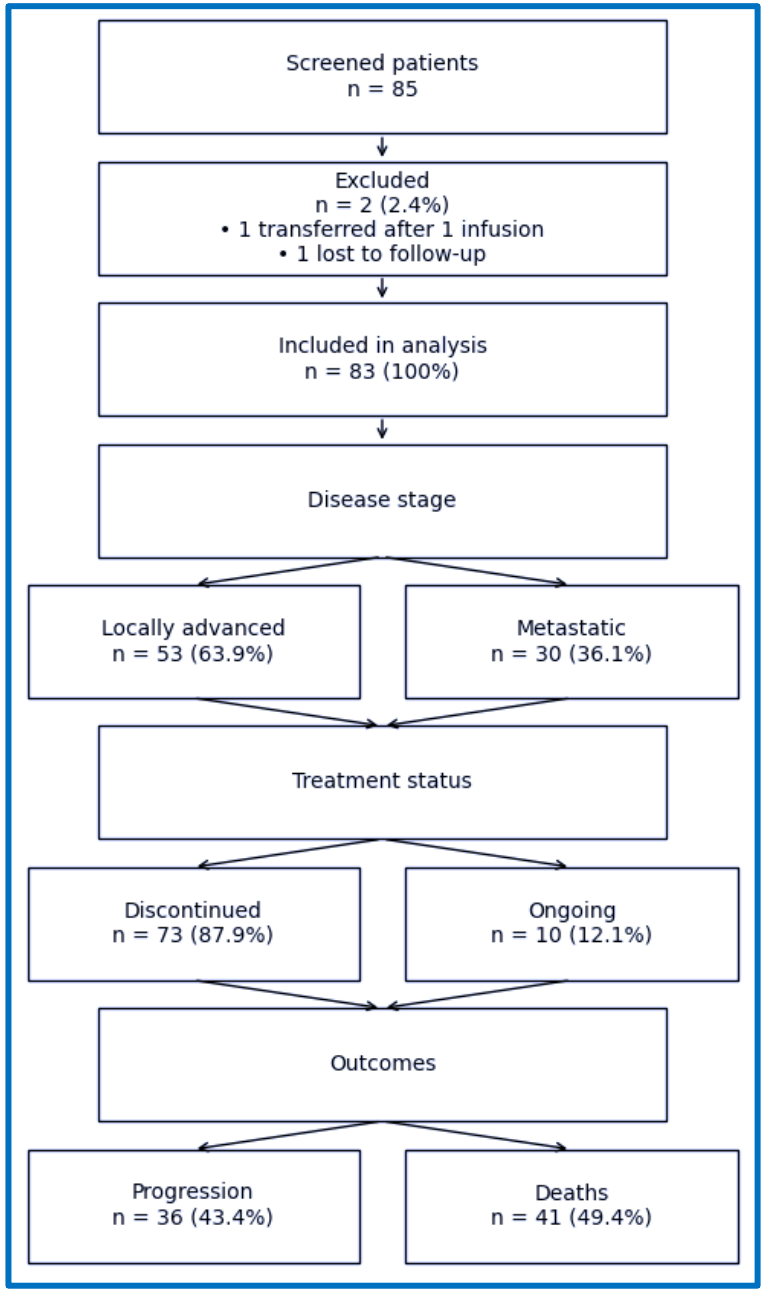
Screened patients
n = 85
Excluded
n = 2 (2.4%)
• 1 transferred after 1 infusion
• 1 lost to follow-up
Included in analysis
n = 83 (100%)
Disease stage
Locally advanced
n = 53 (63.9%)
Metastatic
n = 30 (36.1%)
Treatment status
Discontinued
n = 73 (87.9%)
Ongoing
n = 10 (12.1%)
Outcomes
Progression
n = 36 (43.4%)
Deaths
n = 41 (49.4%)

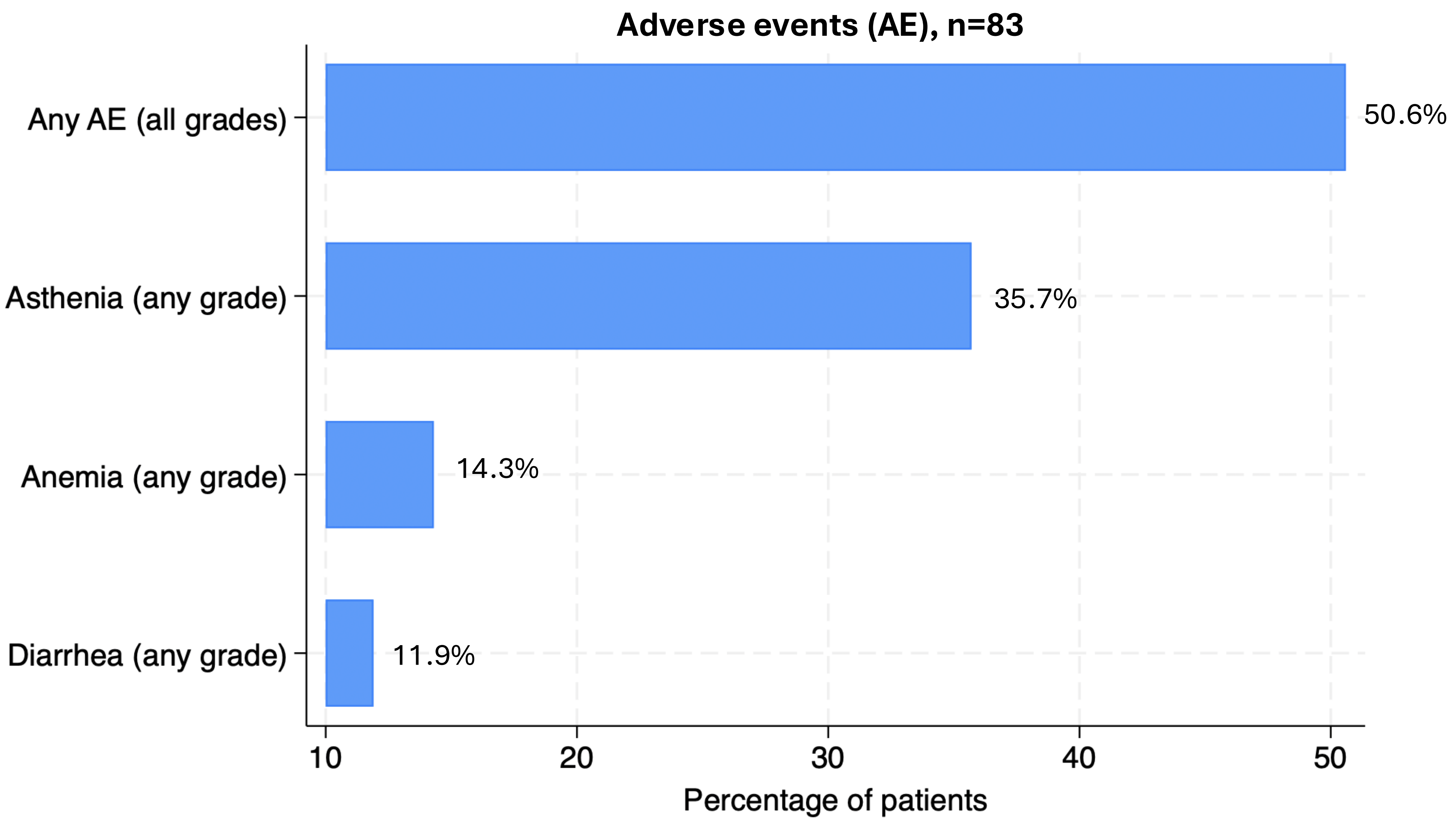

Adverse events (AE), n=83
Any AE (all grades)
Asthenia (any grade)
Anemia (any grade)
Diarrhea (any grade)
50.6%
35.7%
14.3%
11.9%
10
20
30
40
50
Percentage of patients

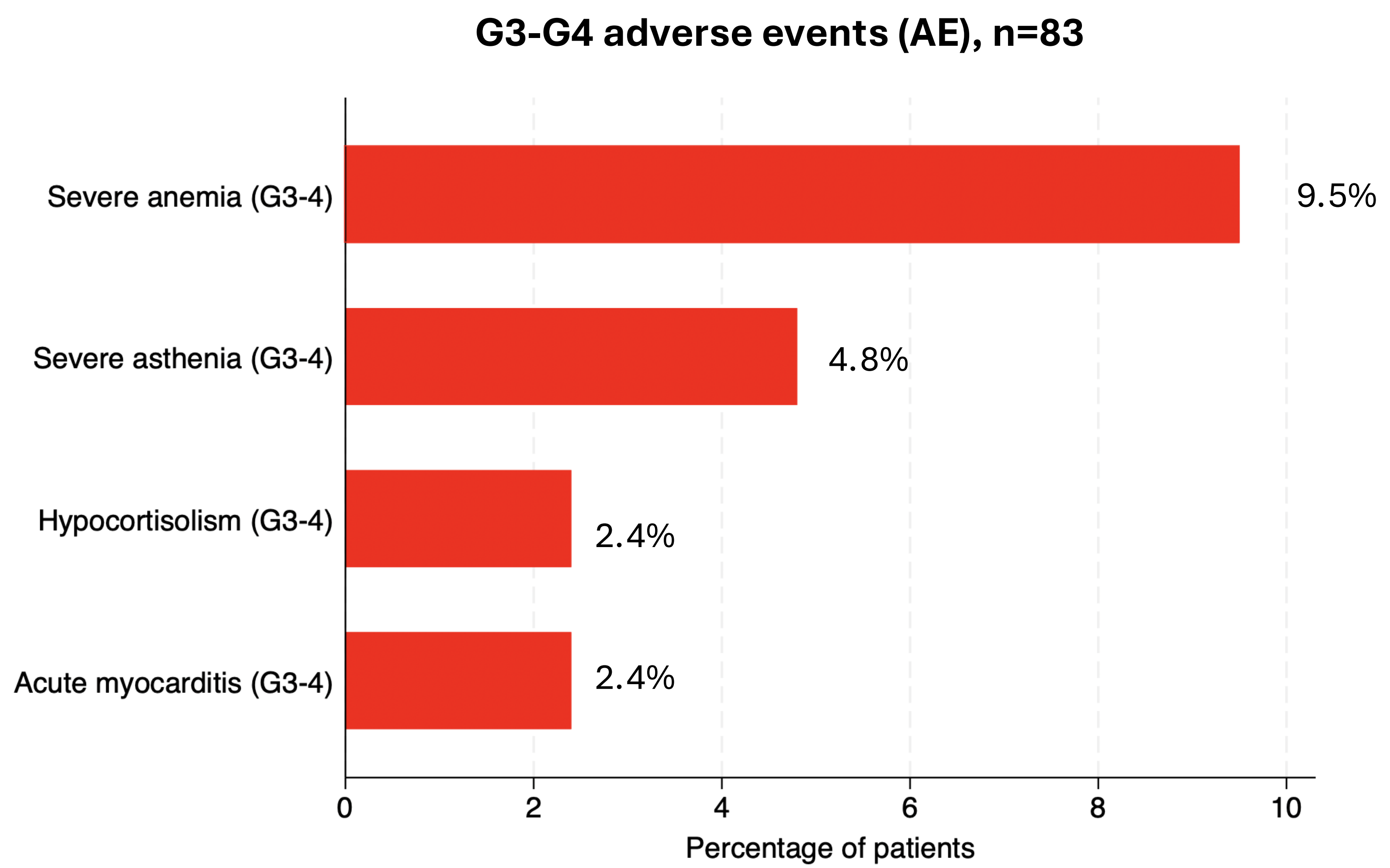
G3-G4 adverse events (AE), n=83
Severe anemia (G3-4)
9.5%
Severe asthenia (G3-4)
4.8%
Hypocortisolism (G3-4)
2.4%
Acute myocarditis (G3-4)
2.4%
0
2
4
6
8
10
Percentage of patients

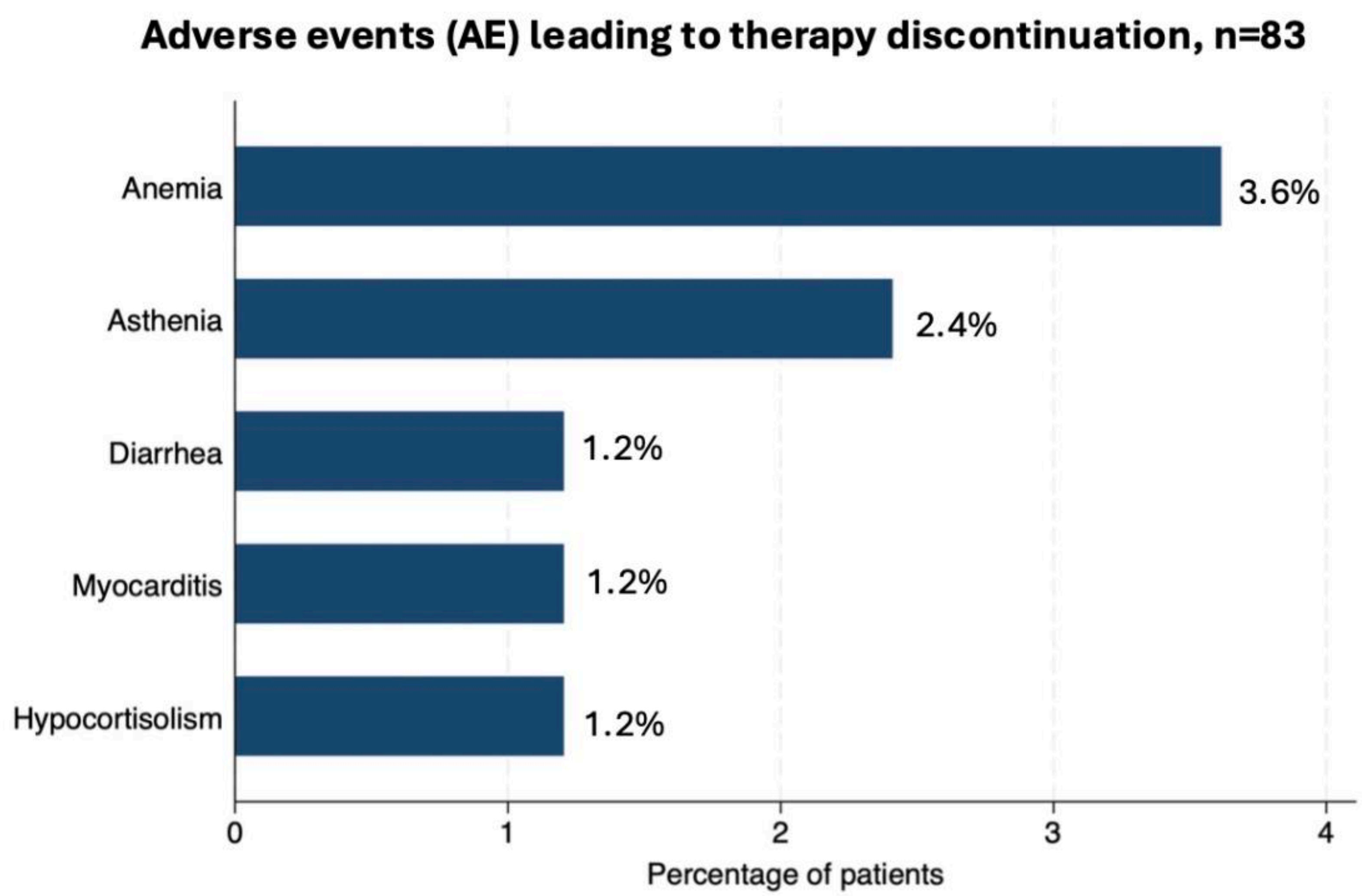
Adverse events (AE) leading to therapy discontinuation, n=83
Anemia
3.6%
Asthenia
2.4%
Diarrhea
1.2%
Myocarditis
1.2%
Hypocortisolism
1.2%
0
1
2
3
4
Percentage of patients
